# The Role of Five‐Membered Aromatic Rings Containing N and O in Modulating Bile Acid Receptors: An Overview

**DOI:** 10.1002/cmdc.202500405

**Published:** 2025-07-11

**Authors:** Claudia Finamore, Carmen Festa, Rosa Barbato, Stefano Fiorucci, Angela Zampella, Simona De Marino

**Affiliations:** ^1^ Department of Pharmacy University of Naples Federico II Via Domenico Montesano 49 Naples 80131 Italy; ^2^ Department of Medicine and Surgery University of Perugia Piazza L. Severi, 1 Perugia 06132 Italy

**Keywords:** five‐membered rings, farnesoid X receptors, G protein‐coupled bile acid receptors 1, heterocyclic compounds, isoxazole

## Abstract

Over the past decades, extensive scientific research in the fields of chemistry and pharmaceutical chemistry has led to the synthesis and study of numerous chemical compounds with diverse therapeutic applications. Many of these compounds feature heterocyclic aromatic structures, including four‐, five‐, and six‐membered rings. Among them, five‐membered heteroaromatic rings have garnered particular attention in medicinal chemistry due to their favorable properties, such as enhanced metabolic stability, solubility, and bioavailability, key attributes for the development of effective drugs. The distinctive physicochemical properties and biological activities of five‐membered heterocycles have established them as vital structural motifs in numerous clinically effective drugs. These heterocyclic compounds play a crucial role in the design of therapeutic agents, including those targeting bile acid receptors. Bile acid receptor modulators, activated by endogenous bile acids, offer promising potential in treating a variety of metabolic and enterohepatic disorders, such as dyslipidemia, diabetes, cholestasis, and inflammatory bowel disease. This review aims to provide an up‐to‐date overview of aromatic five‐membered nitrogen‐ and oxygen‐containing heterocycles, focusing on their role as bile acid receptor modulators, particularly farnesoid X receptor and G protein‐coupled bile acid receptor 1. These receptors are clinically validated targets for the treatment of metabolic disorders and nonalcoholic steatohepatitis.

## Graphical abstract

Several derivatives incorporating five‐membered aromatic rings are described in this review as bile acid receptor modulators, particularly targeting the farnesoid X receptor and the G protein‐coupled bile acid receptor 1. This review provides a comprehensive analysis of patents and literature that is useful to support researchers in the design of new lead compounds for the treatment of liver and metabolic diseases.

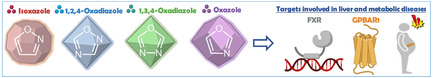

## 1 Introduction

Heterocycles are organic compounds that feature ring structures with at least one atom other than carbon, typically nitrogen (N), oxygen (O), or sulfur (S), within the ring.^[^ ^1^ ^]^

Due to their unique structure, heterocyclic compounds possess a wide range of chemical properties, reactivity, and biological activity, making them fundamental in many fields, including medicine, agrochemistry, and materials science.^[^ ^2^, ^3^, ^4^ ^]^

When considered as a group, heterocyclic derivatives fall into two categories: aromatics and nonaromatics.^[^ ^5^, ^6^ ^]^ Aromatic heterocyclic compounds have a wide range of applications and are predominant among the types of compounds used as pharmaceuticals,^[^ ^7^ ^]^ agrochemicals, and veterinary products.^[^ ^8^, ^9^ ^]^ They are also used as sanitizers, developers, antioxidants, or copolymers and as vehicles in the synthesis of other organic compounds. Many natural products, such as antibiotics penicillins, cephalosporins, and various alkaloids, contain heterocyclic moieties. The most common heterocycles are those with five‐ or six‐membered rings, and among them, five‐membered rings containing nitrogen and oxygen are of significant interest in medicinal and organic chemistry due to their unique electronic properties, reactivity, and broad spectrum of biological activities.^[^ ^10^, ^11^, ^12^, ^13^, ^14^ ^]^ This review focuses on five‐membered rings capable of modulating bile acid receptors (BARs), since these compounds have garnered increasing attention as a promising scaffold in drug discovery. BARs play a central role in regulating bile acid homeostasis, glucose metabolism, lipid metabolism, and inflammatory processes, constituting a heterogeneous family of cell surface and nuclear receptors activated by bile acids.^[^ ^15^, ^16^, ^17^, ^18^ ^]^ Among these, the best‐characterized members are the farnesoid X receptor (FXR),^[^ ^19^, ^20^, ^21^ ^]^ a nuclear transcription factor activated by primary bile acids, and the G protein‐coupled bile acid receptor (GPBAR1), also known as TGR5 or membrane‐bile acid activated receptor (M‐BAR), a seven‐transmembrane G protein‐coupled receptor, activated primarily by secondary bile acids.^[^ ^22^, ^23^ ^]^

Over the past two decades, BARs have been extensively studied for their therapeutic potential in treating various human disorders.^[^ ^24^, ^25^, ^26^, ^27^, ^28^, ^29^, ^30^, ^31^, ^32^, ^33^ ^]^

Among steroidal BARs modulators, the 6‐ethyl‐chenodeoxycholic acid (6‐ECDCA), a FXR agonist commonly known as obeticholic acid (also named OCA or INT‐747),^[^ ^34^ ^]^ has gained significant attention.

Approved by the FDA and EMA in 2016 for the treatment of UDCA‐resistant Primary Biliary Cholangitis (PBC),^[^ ^35^ ^]^ OCA represented a milestone in second‐line therapies for this condition. However, in 2024, the European Commission revoked its approval in Europe for the second‐line PBC treatment due to the emergence of notable side effects, underscoring the importance of balancing efficacy with safety in the development and regulation of novel therapeutic agents. Since the withdrawal of obeticholic acid from the European market, numerous FXR ligands have advanced into clinical trials, though none have yet achieved regulatory approval. The demand for innovative therapeutics remains critical, particularly for addressing widespread conditions such as nonalcoholic fatty liver disease (NAFLD) and its progressive form, nonalcoholic steatohepatitis (NASH).^[^ ^36^ ^]^ NAFLD is a multifaceted condition closely linked to diabetes, dyslipidemia, obesity, and metabolic syndrome.^[^ ^37^ ^]^ Recently reclassified as metabolic dysfunction associated steatotic liver disease (MASLD),^[^ ^38^ ^]^ NAFLD affects nearly 20% of the global population, with its prevalence continuing to rise, particularly in Asian‐Pacific regions. This highlights the urgent need for effective and safe treatment options to manage and mitigate the disease's impact.^[^ ^39^ ^]^ Bile acids and their receptors have been central to therapeutic development for NASH over the last two decades;^[^ ^40^, ^41^ ^]^ however, the failure of OCA in Phase III trials for PBC has exposed a critical limitation of current bile acid‐based therapies, demonstrating the inherent promiscuity of bile acids.^[^ ^42^ ^]^

As a result, there is a growing need for novel and more selective approaches to targeting BARs.

Building on emerging evidence regarding the pharmacological activity of aromatic derivatives with five‐membered rings incorporating nitrogen and oxygen heteroatoms, and inspired by the promising results reported for **GW4064**, a nonsteroidal FXR agonists, and its derivatives,^[^ ^43^, ^44^ ^]^ this review aims to explore the potential of synthetic modulators featuring five‐membered rings for BARs. Particular attention will be given to FXR and GPBAR1 modulators. This novel class of agents is designed for the treatment of metabolic, inflammatory, and hepatic disorders, with the potential to address current therapeutic gaps and overcome limitations of traditional bile acid‐based therapies.^[^ ^45^, ^46^ ^]^

Over the past several years, numerous families of these heterocyclic compounds have been synthesized.^[^ ^44^, ^47^, ^48^ ^]^ Although their pharmacological development is still in its infancy, some of these agents have shown considerable promise. By selectively modulating key pathways involved in disease progression, these compounds could address the shortcomings of traditional therapies.

This targeted approach offers the potential to not only improve efficacy but also minimize adverse effects, paving the way for innovative treatments for MASLD and other complex multifactorial diseases.

## 2 Biological Role and Structural Features of GPBAR1 and FXR

Prior to addressing the core topics of this review, we will briefly introduce the pharmacological targets under consideration. The two main receptors activated by bile acids, GPBAR1 and FXR, are mainly expressed in the gastrointestinal tract, but are also found in the cardiovascular system, immune system, and various endocrine organs.^[^ ^15^, ^49^ ^]^

GPBAR1 is a membrane G protein‐coupled receptor that mediates the nongenomic actions of bile acids, playing a crucial role in energy homeostasis, bile acid and glucose metabolism, and anti‐inflammatory responses. In contrast, FXR is a nuclear receptor that acts as a ligand‐activated transcription factor, regulating the expression of genes involved in bile acid synthesis, lipid metabolism, and detoxification processes.

Structurally, GPBAR1 belongs to the rhodopsin‐like superfamily of G protein‐coupled receptors and features the canonical seven‐transmembrane domain architecture, with its ligand‐binding pocket located within the transmembrane helices. FXR, on the other hand, possesses a conserved ligand‐binding domain (LBD) typical of nuclear receptors, characterized by a hydrophobic cavity capable of accommodating bile acids and various nonsteroidal ligands.

Both receptors can be activated by primary and secondary bile acids, but they also recognize and bind small heterocyclic molecules, including five‐membered aromatic rings. These interactions are mediated through a combination of hydrophobic contacts, hydrogen bonding, and π–π stacking. The presence of heteroatoms in these nuclei further enhances the ligand–receptor interactions by providing additional hydrogen bonding potential and altering electron distribution, which influences the receptor's activation dynamics and regulatory outcomes. The tissue distributions of FXR and GPBAR1^[^ ^50^, ^51^ ^]^ along with their co‐regulatory partners (coactivators and co‐repressors),^[^ ^52^ ^]^ suggest that their modulation could yield synergistic effects, expanding their pharmacological potential, particularly in complex diseases such as NAFLD and NASH.

FXR activation has demonstrated efficacy in several preclinical models of NAFLD/NASH and clinical trials. However, potential side effects observed with FXR agonists have shifted the medicinal chemists’ interest toward the discovery of FXR antagonists, which might be useful for better understanding the function of this receptor. Notably, some studies in animal models indicate that FXR antagonists, rather than agonists, may be more effective in resolving NASH.^[^ ^53^, ^54^, ^55^ ^]^ In addition, FXR antagonists could be used in the treatment of obstructive cholestasis, supported by recent findings that FXR gene ablation protects against liver injury caused by bile duct ligation (BDL).^[^ ^56^ ^]^

A deeper understanding of the structural and functional properties of GPBAR1 and FXR is therefore instrumental for drug design, guiding the development of modulators aimed at generating new therapeutic opportunities for liver pathologies and metabolic diseases, in which these receptors play a pivotal role.

## 3 Five‐Membered Aromatic Rings Containing N and O Modulating FXR and GPBAR1 Receptors

Aromatic five‐membered rings containing N and O are highly prevalent among pharmacologically active compounds, representing versatile “privileged scaffolds” in drug discovery thanks to their chemical modifiability and the ability to strategically decorate the core structure. They play a crucial role in optimizing drug potency and selectivity by serving as bioisosteres and influencing pharmacokinetic and toxicological properties.

In the context of these compounds acting as FXR and GPBAR1 modulators, this review highlights the most effective agents in terms of activation/inactivation across four distinct categories of heterocyclic scaffolds: 1) **1,2‐Oxazole** (also named **Isoxazole**); 2) **1,2,4‐Oxadiazole**; 3) **1,3,4‐Oxadiazole**; 4) **1,3‐Oxazole.**

These scaffolds often function as bioisosteres of classical functional groups, such as carboxylic acids or amide bonds, and can be used to replace labile or metabolically unstable groups. At the same time, their intermolecular interaction profiles allow them to bind to many targets, with weak or moderate affinity. However, their structural versatility permits a wide range of substitutions, enabling the fine‐tuning of biological activity and selectivity. Their favorable chemical stability and synthetic accessibility further enhance their attractiveness as core structures in drug development. Consequently, our work aims to provide a well‐structured collection of helpful information on FXR and GPBAR1 modulators, containing five‐membered heterocycles, facilitating the rational design of new leads for the treatment of metabolic and hepatic diseases.

### 3.1 Isoxazole Nucleus

Isoxazole (or 1,2‐oxazole) represents an important class of five‐membered heterocycles, characterized by an oxygen and a nitrogen atom at adjacent positions. Its significance lies in its broad spectrum of biological activities and therapeutic potential. Therefore, the development of new synthetic strategies and the design of novel isoxazole derivatives should be guided by the latest advancements in research.

**Scheme 1:**
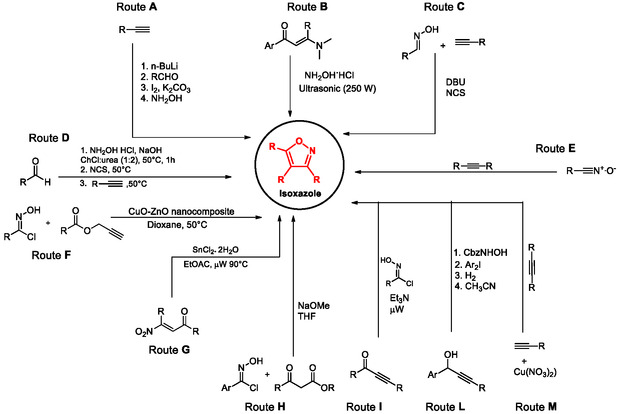
Synthetic routes for the synthesis of 3,5‐ and 3,4,5‐isoxazoles.

Several procedures have been reported in the literature for the synthesis of isoxazoles bearing diverse substituents at positions 3 and 5 or at positions 3, 4, and 5 (**Scheme** **1**).^[^ ^57^ ^]^

Claisen made the first contribution to isoxazole chemistry in 1903 by synthesizing the first compound through the oximation of propargylaldehyde acetal.^[^ ^58^ ^]^ Over the years, several efficient and regioselective methods have been developed for the synthesis of 3,5‐disubstituted isoxazoles,^[^ ^59^, ^60^, ^61^, ^62^, ^63^, ^64^ ^]^ and some of the most recent synthetic procedures are shown below.^[^ ^65^, ^66^ ^]^

Scheme 1 highlights some representative reactions that have been employed since 2014.

#### 3.1.1 Synthesis of 3,5‐Disubstituted Isoxazoles

Various regioselective and efficient strategies have been developed for the synthesis of 3,5‐disubstituted isoxazoles.

A highly regioselective one‐pot method involves the reaction of terminal alkynes with *n*‐BuLi and then aldehydes, followed by the treatment with molecular iodine and subsequently with hydroxylamine (Route **A**, Harigae et al. 2014).^[^ ^67^ ^]^ In the same year, Huang et al. (2014) reported an environmentally benign procedure for the synthesis of 3‐alkyl‐5‐aryl isoxazoles under ultrasound radiation without any catalyst (Route **B**).^[^ ^68^ ^]^ Mohammed and coworkers introduced a metal‐free method using 1,8‐diazabicyclo[5.4.0]undec‐7‐ene (DBU) as a reagent, which promotes the 1,3‐dipolar addition of nitrile oxides with alkynes (Route **C**).^[^ ^69^ ^]^ Pérez et al. (2015) described a sustainable, one‐pot, three‐component synthesis of 3,5‐disubstituted isoxazoles from aldehydes and alkynes, using choline chloride:urea as a deep eutectic solvent (Route **D**).^[^ ^70^ ^]^ In 2016, Kesornpun et al. performed the cycloaddition of nitrile oxides with alkynes, in aqueous solutions under acidic conditions, rather than under the conventional basic conditions (Route **E**).^[^ ^71^ ^]^ More recently, Pardeshi et al. (2022) developed an efficient [3+2] cycloaddition protocol involving in situ generated nitrile oxide from hydroximyl chloride and propargyl ester catalyzed by reusable Cu_2_O–ZnO nanocomposites, without base, under mild conditions and in a short reaction time (Route **F**).^[^ ^65^ ^]^ In 2024, Khan and coworkers realized an efficient method for the conversion of β‐nitroenones into 3,5‐disubstituted isoxazoles.^[^ ^66^ ^]^ The protocol is based on the preliminary reductive reaction of the nitroalkene to the corresponding oxime, which intramolecularly reacts with the carbonyl function, generating the five‐membered ring, which upon dehydration finally affords the isoxazole system (Route **G**).

#### 3.1.2 Synthesis of 3,4,5‐Trisubstituted Isoxazoles

For the synthesis of 3,4,5‐trisubstituted isoxazoles, different strategies have been explored.

The most commonly used is the one reported by Maloney et al. in 2000 for the synthesis of **GW4064** (**1**, Figure 2).^[^ ^43^ ^]^ This approach involves a three‐step reaction, starting from benzaldehyde, following a procedure originally reported by Doyle et al in 1963.^[^ ^72^ ^]^ The key step consists of a condensation reaction between a benzohydroxamoyl chloride, prepared by chlorination of benzaldoxime, and a β‐ketoester. (Route **H**). Alternative procedures involve the use of or in situ generation of a nitrile oxide species.^[^ ^73^, ^74^, ^75^ ^]^ For example, Willy et al. (2008) reported a cycloaddition reaction between an in situ generated nitrile oxide and an alkynone, in the presence of triethylamine, using dielectric heating for 30 min. (Route **I**).^[^ ^76^ ^]^ Gayon et al. (2011) developed a four‐step uninterrupted sequence for the synthesis of trisubstituted isoxazoles from readily available propargylic alcohols, using iron and palladium‐catalyzed systems in succession. This method provided high overall yields and was notably time‐efficient (Route **L**).^[^ ^77^ ^]^ More recently, Li et al. (2017) used syringe pump infusion for the synthesis of polysubstituted isoxazoles using a copper nitrate‐mediated synthesis, from two different alkynes, with high chemo‐and regioselectivity (Route **M**).^[^ ^78^ ^]^

#### 3.1.3 Isoxazole‐Based FXR Agonists

The discovery of **GW4064** (**1**) featuring an isoxazole nucleus as a potent synthetic FXR ligand was made in 2000 by Maloney et al. using a combinatorial chemistry approach.^[^ ^43^ ^]^ Since then, numerous reviews have highlighted the development of isoxazole‐based compounds as FXR agonists.^[^ ^44^, ^47^, ^79^, ^80^ ^]^

**Figure 1:**
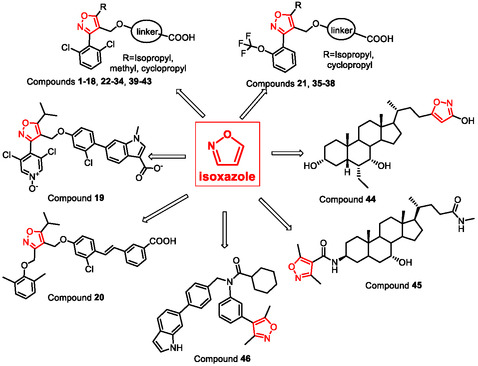
Isoxazole‐based derivatives reported as FXR agonists.

**Figure** **1** summarizes the isoxazole‐based compounds described in this review.

**Figure 2:**
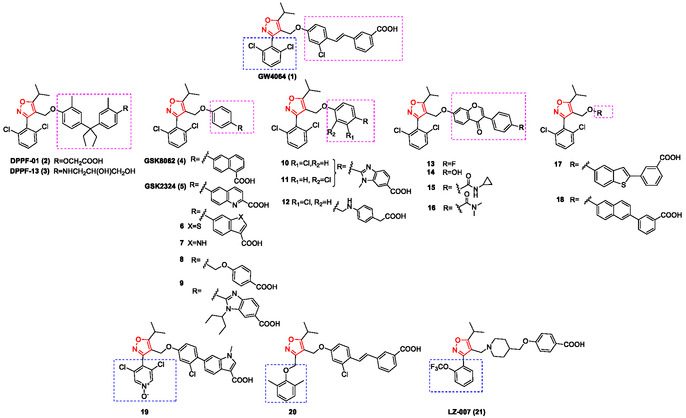
5‐Isopropylisoxazole derivatives reported as FXR agonists.

**Figure** **2** illustrates the most promising synthetic 5‐isopropylisoxazole derivatives reported as FXR agonists from 2006 to the present.

Building on the structural features of chenodeoxycholic acid (**CDCA**), the natural, well‐known steroidal FXR agonist, and **GW4064** (**1**), and considering the potential of diphenylpentane (DPP) as a steroid skeleton substitute, Kainuma et al. in 2006 designed a series of DPP derivatives as FXR ligand candidates (Figure 2).

In **DPPF‐01** (**2**) and **DPPF‐13** (**3**), the stilbene units of **GW4064** (**1**) were replaced with a diphenylmethane structure. **DPPF‐01** (**2**) exhibited significantly stronger and selective agonistic activity toward FXR than the physiological ligand **CDCA** (EC_50_ of 3.4 μM compared to 11.7 μM for **CDCA**; **GW4064** EC_50_ = 15 nM), whereas **DPPF‐13** (**3**) showed lower potency in its agonistic activity than **CDCA**. These findings highlighted the diphenylmethane scaffold as a promising substitute for the steroidal backbone, offering increased specificity and reduced cross‐reactivity with other nuclear receptors (Figure 2).^[^ ^81^ ^]^

Due to the significant limitations presented by **GW4064** (**1**), such as rapid clearance in rats, short terminal half‐life, an oral bioavailability <10%, potential toxicity and instability under UV light ascribable to its stilbene functionality,^[^ ^82^, ^83^ ^]^ in 2008, Akwabi‐Ameyaw et al. prepared a series of stilbene replacements,^[^ ^84^ ^]^ maintaining the structural rigidity necessary for FXR activation. Their design strategy involved the replacement of the stilbene unit with a naphthalene ring bearing the carboxylic moiety at position 6, as exemplified by **GSK8062** (**4**, Figure 2). This compound demonstrated good FXR agonistic activity in fluorescence resonance energy transfer (FRET) and in transient transfection (TT) assays (FRET EC_50_ = 87 nM; TT EC_50_ = 68 nM), equivalent to **GW4064**, but with improved pharmacokinetic properties. Additionally, it reduced cholestatic effects in an ANIT‐induced rat model of acute cholestasis, highlighting its potential as a promising candidate for drug development.

In 2011, Bass et al.^[^ ^85^ ^]^ developed a series of analogs to enhance the properties of **GSK8062** (**4**),^[^ ^84^ ^]^ including the quinoline‐based compound **GSK2324** (**5**, Figure 2). **GSK2324** (**5**) demonstrated equipotent FXR agonist activity (FRET EC_50_ = 120 nM; TT EC_50_ = 50 nM) to **GSK8062** (**4**) but with significantly enhanced pharmacokinetic properties, including a fivefold increase in oral bioavailability in rats and improved solubility. It also showed a favorable cytochrome P450 (Cyp450) inhibition profile and high permeability in absorption assays. In a diet‐induced obese mouse model, **GSK2324** (**5**) reduced body weight gain and serum glucose levels, accompanied by decrease in triglycerides and total cholesterol. Its improved pharmacokinetic profile and dose‐dependent metabolic benefits suggested that this compound was a promising candidate for further investigation into FXR modulation and related therapeutic applications. To further explore the optimal placement of the carboxylic moiety in conformationally constrained analogs of **GW4064**, Akwabi‐Ameyaw et al.^[^ ^86^ ^]^ synthesized several stilbene replacements. The benzothiophene (**6**) and indole analogs (**7**) (Figure 2) revealed that the ideal orientation of the carboxylic group is coplanar with its ring, forming a 150° angle with the linking phenyl ring, an arrangement that enhances FXR agonist potency. Compounds **6** and **7** showed high selectivity and potency for FXR (>100‐fold; **6** FRET EC_50_ = 21 nM, TT EC_50_ = 24 nM and **7** FRET EC_50_ = 20 nM, TT EC_50_ = 31 nM) over other nuclear receptors, such as LXRα, LXRβ, PPARα, γ, δ, as well as PXR, and exhibited low inhibition of Cyp450 enzymes (>5000 nM). Despite their promising selectivity and potency, these analogs displayed poor pharmacokinetic profiles, limiting their potential as oral agents. Nonetheless, they provide valuable insights for designing improved FXR modulators.

In 2019, Sepe et al. reported the identification of novel isoxazoles with FXR agonistic activity, also capable of preventing liver injury.^[^ ^87^ ^]^ Starting from **GW4064**, several modifications were introduced on the oxymethylene group at C‐4, while preserving the central isoxazole core, the isopropyl group at C‐5, and the 2,6‐dichloro‐substituted phenyl moiety at C‐3. The efficacy of these compounds was initially evaluated using a *cell‐free* AlphaScreen assay and subsequently confirmed through a transactivation assay on HepG2 cells. Compound **8** (Figure 2) emerged as the most potent derivative (EC_50_ = 0.30 ± 0.006 μM, efficacy = 149%), exhibiting favorable pharmacokinetic properties and showing a remarkable ability to bind and regulate FXR activity in vivo. Furthermore, it successfully rescued mice from acute liver failure caused by acetaminophen overdose in an FXR‐dependent manner.

Masuda et al. (2020), in order to selectively activate FXR, designed and synthesized a series of N1‐substituted benzimidazole‐based compounds.^[^ ^88^ ^]^ Starting from a previous work, in which they reported a new chemotype of FXR agonists,^[^ ^89^ ^]^ they focused attention on enhancing FXR agonistic potency. Among the synthesized library, compound **9** (Figure 2) demonstrated a strong FXR agonistic activity, with an EC_50_ of 26.5 ± 10.5 nM in time‐resolved fluorescence energy transfer (TR‐FRET) assay and 0.8 ± 0.2 nM in luciferase transactivation assay.

Additionally, compound **9** (Figure 2) promoted differentiation of ST2 cells (murine bone marrow‐derived mesenchymal stem cell‐like cells) into osteoblasts, suggesting a potential role in bone metabolism regulation.

In continuation of the efforts, novel FXR agonists, featuring isoxazole and N‐substituted benzimidazole moieties, were synthesized by Fujimori et al. in 2019. They compared the effects on osteoblast differentiation of the synthesized compounds with the known FXR agonists, chenodeoxycholic acid (**CDCA**) and **GW4064**.^[^ ^89^ ^]^ Compounds **10** and **11** (Figure 2) demonstrated high specificity for FXR, showing a FRET EC_50_ of 13.4 ± 7.9 nM for compound **10** and 31.6 ± 26.9 nM for compound **11** and computer‐assisted modeling studies demonstrate that **10** binds to the same FXR‐LBD binding site as **GW4064** (**1**). The study revealed a correlation between FXR activation and mesenchymal stem cell differentiation, promoting the early stage of BMP‐2‐induced osteoblast differentiation. These findings suggest that FXR could be a promising therapeutic target for bone diseases like osteoporosis.

A promising multitarget approach for treating metabolic diseases by combining peripheral PPARδ‐mediated activity with the hepatic effects of FXR activation was proposed by Schierle et al. (2020).^[^ ^90^ ^]^ They designed a minimal dual FXR/PPARδ activator scaffold by rationally merging pharmacophores from existing selective agonists. This approach resulted in the development of compound **12** (Figure 2), which exhibits high and balanced potency on both nuclear receptors (EC_50_ of 0.94 ± 0.07 μM for FXR *versus* EC_50_ 1.5 ± 0.2 μM for PPARδ) while demonstrating exceptional selectivity over related transcription factors. In 2018, Qiu et al. designed and synthesized isoflavone derivatives aimed at reducing lipid accumulation.^[^ ^91^ ^]^ Building on their previous work,^[^ ^92^, ^93^ ^]^ which demonstrated the antidyslipidemic properties of isoflavone compounds, they developed a series of isoflavone hybrid compounds combined with **GW4064** (**1**). Many of these compounds effectively decreased lipid buildup in 3T3‐L1 adipocytes, with compounds **13‐16** (Figure 2) exhibiting greater inhibitory effects than **GW4064** (**1**). The most potent compound **16** not only acted as a promising agonist for FXR in cell‐based assays but also modulated gene expression by up‐regulating FXR, SHP, and BSEP while down‐regulating SREBP‐1c, a gene associated with lipogenesis.

Compared to **GW4064**, compound **16** showed an improved safety profile in HepG2 cytotoxicity assays. Molecular docking studies further confirmed that **16** fits well within the FXR binding pocket, interacting with key residues.

Previously, Akwabi‐Ameyaw et al. (2009) reported the synthesis of two series of **GW4064** (**1**) analogs, aiming to improve metabolic stability by replacing the metabolically labile stilbene structure. The stilbene was substituted with either benzothiophene or naphthalene rings, leading to the identification of potent full agonists, specifically compounds **17** and **18** (EC_50_ = 55 nM and 110 nM, respectively).^[^ ^94^ ^]^

In 2009, Feng et al. developed a series of 3‐aryl heterocyclic isoxazole derivatives based on docking studies and co‐crystallographic analysis of **GW4064** (**1**) bound to FXR.^[^ ^95^ ^]^ Among these compounds, the N‐oxide pyridine analog (**19,** Figure 2) stood out for its remarkable FXR activity. This analog, inspired by Eli Lilly's patent^[^ ^96^ ^]^ involving phenyl indole as a substitute for stilbene, exhibited the strongest binding affinity for FXR at 0.045 μM, achieved an efficacy of 53.7%, and demonstrated significantly improved physicochemical properties compared to the other derivatives. Bass et al. (2009) synthesized a series of alternately 3,5‐substituted isoxazoles based on the known FXR agonist **GW4064**. Several analogs demonstrated potent FXR agonistic activity, with compounds featuring a linker between the isoxazole ring and the 3‐aryl substituent showing potency comparable to **GW4064**. Notably, the 2,6‐dimethyl phenol analog (**20**, Figure 2) displayed greater potency in FXR assay (FRET EC_50_ = 17 nM; TT EC_50_ = 56 nM) than **GW4064** itself highlighting the potential of structural modifications in the isoxazole scaffold to enhance FXR agonistic activity and contributing to the development of more effective FXR‐targeted therapies.^[^ ^97^ ^]^

A novel series of FXR agonists, featuring a piperidine scaffold, was designed by Wang et al. (2025) to reduce the high lipophilicity of existing FXR agonists^[^ ^98^ ^]^ Through a comprehensive multiparameter optimization of compound **8** previously reported,^[^ ^87^ ^]^ they identified **LZ‐007** (**21**, Figure 2), as highly potent FXR agonist (FRET EC_50_ = 51 ± 10 nM; TT EC_50_ = 76 ± 17 nM), with suitable stability in liver microsomes across multiple species. **LZ‐007** (**21**) exhibited high oral bioavailability and selective tissue distribution in the liver and ileum, while maintaining low plasma exposure, potentially minimizing systemic side effects; it significantly upregulated FXR and its downstream genes in these target tissues. In the CCl_4_‐induced MASH model, **LZ‐007** (**21**) effectively improved multiple pathological indicators by modulating key signaling pathways related to lipid metabolism, oxidative stress, inflammation, and fibrosis, showing in a 30‐day toxicity study no apparent adverse effects, even at high doses. With its promising pharmacodynamic and safety profile, **LZ‐007** (**21**) emerges as a strong candidate for further evaluation as a potential anti‐MASH agent.

**Figure 3:**
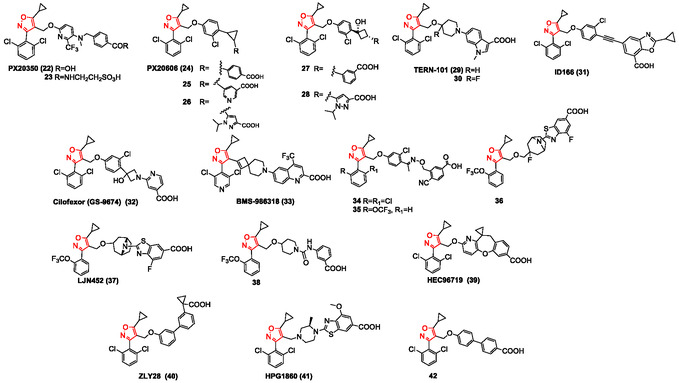
5‐Cyclopropylisoxazole derivatives reported as FXR agonists.

**Figure** **3** illustrates the 5‐cyclopropylisoxazole derivatives reported in this review as FXR agonists.

Abel et al. (2010) described the synthesis and pharmacological evaluation of a novel series of FXR agonists, designed to maintain the core features of **GW4064** (**1**) but replacing its *trans*‐stilbene moiety with 3‐ and 4‐benzoic acid moieties linked to a substituted pyridine via oxymethylene or amino‐methylene linkers.^[^ ^99^ ^]^ These structural modifications led to compounds with enhanced in vitro potency. A key feature of this series is the presence of either a free terminal benzoic acid or its amide conjugate, which extends the acidic moiety without significantly reducing activity in both biochemical and cellular FXR assays. In vivo studies demonstrated that compound **22,** then renamed **PX20350** by Phenex (FRET EC_50_ = 12 nM, Figure 3),^[^ ^100^ ^]^ induced a dose‐dependent reduction in total plasma triglycerides and cholesterol levels. Interestingly, this nonsteroidal FXR agonist undergoes metabolic transformation, including taurine and glucuronic acid conjugation, similar to natural bile acids. Compound **23**, a metabolite of **PX20350** (**22**), acted as a direct FXR ligand and a substrate of Na^+^‐taurocholate cotransporting polypeptide (NTCP), as shown in functional studies. However, compounds **22** and its derivative **23** were not detected in plasma or liver at significant concentrations at this time point. This suggests a lower risk of prolonged off‐target effects, while still providing a sufficient pharmacodynamic duration to impact lipid homeostasis.

In 2012, Hambruch et al. investigated the effects of synthetic FXR agonists on cholesterol metabolism and atherosclerosis prevention,^[^ ^101^ ^]^ demonstrating that different FXR agonists are able to induce variations in pharmacological effects on liver gene expression and the clearance of HDL‐derived cholesterol into feces. In a transgenic mouse model lacking low‐density lipoprotein receptors (LDLR^−/−^), which are prone to developing atherosclerosis, treatment with FXR agonists led to a significant reduction of atherosclerotic lesions, suggesting that FXR activation can help to mitigate the development of atherosclerosis, a condition characterized by the accumulation of cholesterol‐rich plaques in arterial walls. Among the FXR agonists examined were the isoxazole derivative **GW4064** (**1**) and two structurally related compounds, **PX20350** (**22**),^[^ ^99^ ^]^ previously reported, and **PX20606** (**24**, FRET EC_50_ = 32 nM) (Figure 3).^[^ ^102^ ^]^ Additionally, two weeks of treatment with **PX20606** (**24**) resulted in a significant reduction in plasma total cholesterol and triglyceride levels, along with a strong, dose‐dependent reduction in aortic plaque formation. To improve absorption, distribution, metabolism, and excretion (ADME) properties of **GW4064** (**1**), Kinzel et al. in 2016, synthesized novel compounds featuring cyclopropyl, hydroxycyclobutyl, or hydroxyazetidinyl linkers, connecting the “hammerhead” isoxazole to a terminal aryl or heteroaryl group, bearing a carboxylic acid.^[^ ^103^ ^]^ All synthesized compounds maintained high FXR potency but exhibited distinct physicochemical and pharmacokinetic profiles. The carboxylic acid position on the aromatic ring also had a notable effect on pharmacokinetics, with the hydroxycyclobutyl and azetidinyl linkers leading to better pharmacokinetic properties than cyclopropyl. Pharmacodynamic assessments in high‐fat‐diet‐fed mice demonstrated significant plasma lipid profile improvements, with compounds **25‐28** (FRET EC_50_ = 10, 7, 29, and 9 nM, respectively) (Figure 3) showing marked cholesterol reductions. The introduction of hydroxyl‐bearing 4‐membered rings (compounds **27** and **28**) as linkers successfully reduced amphiphilicity and improved polarity while maintaining FXR agonist potency, offering favorable drug‐like properties despite the lack of a direct correlation between physicochemical and pharmacokinetic/pharmacodynamic properties.

In 2015, Genin et al. developed a series of potent FXR agonists to treat dyslipidemia, leading to the identification of **LY2562175**, now named **TERN‐101** (**29**, Figure 3), a selective FXR agonist with strong lipid‐modulating properties (TT EC_50_ = 193 nM).^[^ ^104^ ^]^ In preclinical models, **TERN‐101** (**29**) reduced LDL and triglycerides while increasing HDL levels, showing promising pharmacokinetics (PK) for once‐daily dosing. In insulin‐resistant ZDF rats, it significantly reduced triglycerides and raised HDL by up to 95%, supporting its advancement into human clinical trials. A phase I study assessed the safety, PK, and pharmacodynamics (PD) of **TERN‐101** (**29**) in healthy volunteers.^[^ ^105^ ^]^ In a randomized, double‐blind, placebo‐controlled trial, 38 participants received **TERN‐101** capsules (25, 75, or 150 mg) for 7 days, while an open‐label study evaluated single doses of **TERN‐101** (5 and 25 mg tablets and 25 mg capsules). **TERN‐101** (**29**) was well tolerated, with no cases of pruritus reported, supporting its further evaluation for NASH treatment.

Li et al. reported in 2020 a series of nonbile acid FXR agonists designed and synthesized based on a structure‐based drug design and structural optimization strategies.^[^ ^106^ ^]^ Among the 36 synthesized compounds, compounds **30** and **36** (Figure 3) demonstrated prominent PK profiles and high selectivity for FXR (5.8 nM and 8.7 nM, respectively) without activation on GPBAR1. Notably, compound **36** significantly improved liver injury markers (AST, ALT), reduced fibrosis progression, and effectively improved steatosis, inflammation, injury, and fibrosis in the diet‐combined chemical‐induced mice NASH model, which is superior to clinical drugs like OCA. These results support compound **36** as a promising candidate for further exploration in FXR‐dependent disease therapies.

Compound **ID166** (**31**) was designed by Moon et al. in 2024, with a triple bond linker and a terminal benzoxazole moiety maintaining the well‐known trisubstituted isoxazole “hammerhead” structure.^[^ ^107^ ^]^ This compound demonstrated potent FXR agonist activity in vitro, with an EC_50_ of 3 nM in TR‐FRET and 5 nM in the FXR gene reporter assay. Furthermore, **ID166** (**31**) effectively induced SHP gene expression in primary human hepatocytes (EC_50_ = 19 nM), showing superior efficacy compared to the clinical reference compound OCA. It exhibits high selectivity for FXR and in vivo studies demonstrated that oral administration in hamsters and mice significantly upregulated FXR target genes in a dose‐dependent manner, with a preference for intestinal distribution over liver accumulation. **ID166** (**31**) also improved histological features of NASH and reduced liver fibrosis in multiple animal models. Its unique pharmacological profile suggests it could be an effective therapeutic candidate for NASH, offering enhanced efficacy while minimizing side effects like liver injury and pruritus.

Cilofexor (also known as **GS‐9674** (**32**), Figure 3) was reported by Trauner et al. (2019) in a randomized, placebo‐controlled study to determine its safety and efficacy in patients with primary sclerosing cholangitis (PSC) without cirrhosis.^[^ ^108^ ^]^ Cilofexor (**32**) had previously demonstrated anti‐inflammatory and antifibrotic effects (EC_50_ = 43 nM), as well as a reduction in portal pressure in preclinical models of liver fibrosis, as shown by Schwabl et al. (2018).^[^ ^109^ ^]^ In this phase II, randomized, double‐blind, placebo‐controlled trial, 52 patients were treated for 12 weeks with Cilofexor (**32**) (100 mg, 30 mg) or placebo. Treatment with 100 mg led to significant reductions in transaminase levels, along with improvements in liver biochemistry and cholestasis markers. Adverse events were similar across groups, with pruritus being more common in placebo‐treated patients. The results demonstrated that Cilofexor (**32**) was well tolerated and showed potential benefits for PSC.

Carpenter et al. identified a series of spirocyclic‐containing compounds, notably **BMS‐986 318** (**33**, Figure 3), which showed strong in vitro activation of FXR, favorable ADME properties, and in vivo efficacy.^[^ ^110^ ^]^ Targeting the methylene ether linkage to improve ADME properties, while maintaining FXR agonist potency, they synthesized and tested new compounds in FXR luciferase and steroid receptor coactivator 1 (SRC‐1) recruitment assays.^[^ ^111^ ^]^ Derivative **BMS‐986 318** (**33**), despite being lower in vitro potency (EC_50_ = 53 nM) and plasma exposure compared to other analogs, induced comparable gene changes and demonstrated efficacy in a mouse model of liver cholestasis and fibrosis. These results suggest its potential as an antifibrotic therapy worthy of further investigation.

In the effort to develop nonsteroidal FXR agonists for the treatment of NASH and other bile acid‐related diseases, a 2021 study^[^ ^112^ ^]^ reported the synthesis of a series of novel compounds based on modifications of **GW4064** (**1**), specifically replacing its stilbene group with a ketoxime ether moiety. Although significant progress has been made in manipulating the **GW4064** scaffold to enhance druggability, no FXR agonist has yet been approved for NASH treatment.^[^ ^84^, ^85^, ^103^, ^104^, ^113^, ^114^, ^115^ ^]^ Among the synthesized compounds, **34** and **35** (Figure 3) exhibited potent FXR agonistic activities in vitro (EC_50_ = 45.2 ± 19.5 nM and 36.1 ± 4.7 nM, respectively) and effectively promoted the expression of target genes in vivo. Notably, these compounds displayed some favorable properties, including oral effectiveness and high liver–to–blood distribution ratios, which could help minimize potential side effects associated with prolonged systemic FXR activation.

In 2021, Luo et al., in order to identify new isoxazole‐based FXR agonists with a nonsteroidal core, discovered a series of derivatives bearing aryl urea moieties through the structural simplification of **LJN452** (**37**, Figure 3), which is currently in Phase 2 clinical trials.^[^ ^115^ ^]^ Compound **38** emerged as a potent FXR agonist, exhibiting similar agonistic potency but lower maximum efficacy compared to the full agonists **GW4064** and **LJN452** (**37** FRET EC_50_ = 0.20 nM) in a cell‐based FXR transactivation assay.^[^ ^116^ ^]^ Extensive in vitro evaluations further confirmed the partial efficacy of **38** (EC_50_ = 0.41 μM) in FXR‐dependent gene modulation and revealed its lipid‐reducing activity. More importantly, oral administration of **38** in mice demonstrated favorable pharmacokinetic properties, leading to promising in vivo FXR agonistic activity.

Overall, compound **38** represented a promising new scaffold for FXR modulators, warranting further investigation into the treatment of FXR‐dependent metabolic and cardiovascular diseases.

**HEC96719** (**39**, Figure 3),^[^ ^117^ ^]^ a novel tricyclic FXR agonist derived from **GW4064** reported by Cao et al. demonstrated a 150‐fold increase in potency compared to **GW4064** and OCA, with improved bioavailability and stability (**39** FRET EC_50_ = 1.37 ± 0.31 nM). Their design strategy involved replacing the reactive alkene moiety with a saturated group while maintaining structural rigidity. Initial compounds showed metabolic instability due to a dihydroquinone moiety, which was addressed by incorporating a nitrogen atom to prevent toxic benzoquinone formation. Further optimization led to **HEC96719** (**39**), which showed a 66‐fold increase in activity. **HEC96719** exhibited high liver and ileum distribution, effectively sustaining FXR activation and FGF15/19 expression. It also demonstrated superior efficacy in preclinical NASH and liver fibrosis models compared to OCA. Overall, preclinical data suggest **HEC96719** as a promising drug candidate for NASH treatment.

Aiming to optimize therapeutic strategies for NASH, a 2023 study by Ren et al. focused on the development of a dual‐target modulator able to activate FXR and to inhibit FABP1. The researcher designed hybrid molecules based on known clinical compounds, with better inhibitory activity against FABP1.^[^ ^118^ ^]^ Through comprehensive multiparameter optimization studies, they identified **ZLY28** (**40**, Figure 3) as a promising FXR/FABP1 dual modulator with suitable stability of liver microsomes and high target selectivity. Interestingly, **ZLY28** revealed a distinct tissue distribution property, with reduced systemic exposure that may translate into better safety by decreasing both on‐ and off‐target side effects. In NASH models, **ZLY28** exerted robust anti‐NASH effects by inhibiting FABP1 and activating the FXR‐FGF15 signaling pathway in the ileum (EC_50_ = 491 nM), impacting key processes such as lipid metabolism, inflammation, oxidative stress, and fibrosis. Based on its overall pharmacological, tissue distribution, and preliminary safety profiles, **ZLY28** has advanced to preclinical study as the first‐in‐class intestinal restricted FXR/FABP1 dual modulator for the treatment of NASH/NAFLD.

In 2023, Mo et al. described the design, optimization, and characterization of a series of nonbile acid FXR agonists based on the known **GW4064**.^[^ ^119^ ^]^ By analyzing structural data and binding interactions between **GW4064** and the FXR‐LBD, they identified the N‐methylene‐piperazinyl motif as a key modification, while preserving the characteristic isoxazole scaffold. Among the compounds developed, compound **41** (**HPG1860**, Figure 3) emerged as a potent full FXR agonist (EC_50_ = 0.018 μM). It demonstrated high selectivity, a favorable in vitro profile, and desirable pharmacokinetic properties. It significantly influenced the transcription of target genes in a rodent PD model and demonstrated superior efficacy over the positive control OCA in a commonly used NASH model. Compound **41** (**HPG1860**) is currently in phase II clinical development for patients with NASH.

Recently, Jiao et al. reported a two‐round high‐throughput screening that led to identify a new FXR agonist as a nonphenolic 17‐β‐hydroxysteroid dehydrogenase 13 (HSD17B13) inhibitor.^[^ ^120^ ^]^ The HSD17B13 is a liver‐specific protein linked to lipid metabolism and chronic liver diseases, and it is upregulated in MAFLD.^[^ ^121^, ^122^ ^]^ Structural optimization led to the discovery of compound **42**, a dual FXR/HSD17B13 modulator (FXR EC_50_ = 52 nM; HSD17B13 IC_50_ = 0.27 μM), with favorable tissue distribution and suitable pharmacokinetic properties (Figure 3). This compound demonstrated superior therapeutic effects compared to OCA in multiple MASH models, even at lower doses. It displayed high oral bioavailability (F = 46%) and a half‐life of 5.3 h, with broad therapeutic effects against metabolic syndrome, including weight gain prevention, hepatic steatosis reduction, and glucose/lipid metabolism improvement. Based on its promising pharmacological and safety profile, compound **42** has advanced to preclinical studies for MASH treatment.^[^ ^123^ ^]^

**Figure 4:**
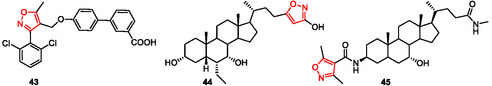
5‐Methylisoxazole derivative (**43**) and bile acids‐isoxazole hybrids **44** and **45**.

Rapacciuolo et al. reported the discovery of a novel class of hybrid molecules with dual activity toward FXR and the leukemia inhibitory factor receptor (LIFR), harnessing the 3,4,5‐trisubstituted isoxazole scaffold.^[^ ^124^ ^]^ Through the pharmacological characterization of 27 designed and synthesized derivatives, compound **43** (**Figure** **4**) was identified as a potent FXR agonist and LIFR antagonist with excellent ADME properties (FXR EC_50_ = 3.2 μM, LIF‐LIFR IC_50_ = 2.0 μM). In vitro and in vivo studies established compound **43** as the first‐in‐class orally active hybrid LIFR inhibitor and FXR agonist, demonstrating protective effects against the development of acute liver fibrosis and inflammation.

In 2017, Xiao et al. reported a series of novel bile acids featuring various modifications on the A‐ring and side chain of OCA, with the aim to reduce adverse effects (e.g., pruritus and increased LDL levels) potentially associated with dual activity on GPBAR1.^[^ ^125^ ^]^ The best lead identified in this study, **compound 44** (Figure 4), characterized by a 3,5‐disubstituted isoxazole moiety, bearing a hydroxyl group at the 3‐position, demonstrated high efficacy on FXR (EC_50_ = 0.03 μM, efficacy = 121%), with a potency six times greater than that of OCA and an EC_50_ ratio of 17 between FXR and GPBAR1. This compound represents one of the few examples of a disubstituted isoxazole FXR agonist reported in the literature.

In 2018, in a drug discovery program for hyperlipidemia, Qiu et al. designed, synthesized, and evaluated a series of novel isoxazole–CDCA hybrid molecules as antilipidemic agents.^[^ ^126^ ^]^ All ten hybrids synthesized in their work reduced lipid accumulation in 3T3‐L1 adipocytes at a concentration of 10 μM, with compound **45** (Figure 4), achieving the highest reduction of 30.5%, without toxicity. The isoxazole group was linked to CDCA at the C‐3 position via an amide bond, inspired by treatments for metabolic syndrome like Aramchol, while modifications at the C‐24 side chain led to the identification of **45** as the most effective derivative.

Mechanistic studies in HepG2 cells revealed that **45** activated the FXR‐SHP signaling pathway and downregulated SREBP‐1c, a key lipogenesis regulator. This compound exhibited binding patterns similar to the FXR agonist **GW4064** and showed no cytotoxicity at 10 μM, demonstrating its potential as a promising lipid‐lowering agent through FXR activation and regulation of lipid metabolism.

**Figure 5:**
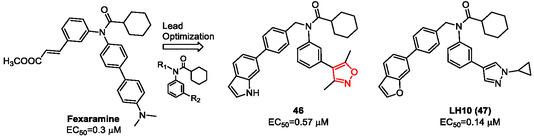
Fexaramine derivatives **46** and **47**.

Fexaramine served as a starting point for the development of a series of FXR agonists aimed at treating liver diseases, as reported by Huang et al. in 2024.^[^ ^127^ ^]^ These compounds were strategically developed to improve efficacy and selectivity, addressing the significant side effects reported in clinical trials for most existing FXR agonists. In this study, they reported the synthesis of **LH10** (**47,** **Figure** **5**), a potent FXR agonist featuring a cyclopropylpyrazole group at the R_2_ position on the fexaramine scaffold, along with several other derivatives. Among these, compound **46**, endowed with an isoxazole nucleus, displayed good binding affinity for FXR, although it was less potent than fexaramine (EC_50_ = 0.3 μM for fexaramine vs. EC_50_ = 0.57 μM for compound **46**).

#### 3.1.4 Isoxazole‐Based FXR Antagonists

Different isoxazole derivatives have been proven to be effective as FXR antagonists. The development of FXR ligands, particularly nonsteroidal antagonists, is highly significant for dissecting and shedding light on the complex biological pathways under FXR control and for understanding the molecular‐level conformational changes of the receptor in its agonistic and antagonistic forms.

In recent years, the consideration of OCA side effects in clinical trials, which represents the most promising steroidal FXR agonist, together with a recent finding that the FXR gene ablation protects from liver injury caused by BDL,^[^ ^36^ ^]^ have moved the scientific attention toward the FXR antagonism for the treatment of obstructive cholestasis.^[^ ^56^ ^]^

**Figure 6:**
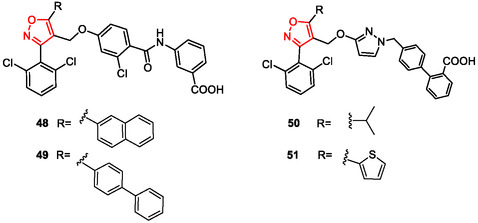
Isoxazole‐based FXR antagonists **48–51**.

Starting from 2007, chemical modifications at the 5‐position of the isoxazole ring played a crucial role in modulating antagonistic potency as reported by Kainuma et al.^[^ ^128^ ^]^ Among the compounds studied, compounds **48** and **49** (**Figure** **6**), featuring 2‐naphthyl and 4‐biphenyl substituents, respectively, emerged as potent FXR antagonists with higher selectivity than the naturally occurring FXR antagonist guggulsterone (IC_50_ = 4.0 μM and 3.7 μM, respectively, versus 12 μM for guggulsterone). Similarly, starting from the previously reported FXR antagonists characterized by a trisubstituted isoxazole scaffold, Huang et al. (2015) designed a new library of nonsteroidal FXR antagonists (compounds **50** and **51**) as potential conductors for further structural modification studies.^[^ ^129^ ^]^ The authors proposed a key mechanism for FXR antagonism, identifying residue E467 on helix H12 of FXR as a crucial hotspot. This residue may play an important role in the antagonistic activity of these compounds, providing valuable insight for future drug design and optimization in FXR‐targeted therapies. Among the tested compounds, the most potent antagonist, compound **51**, was used in molecular dynamics simulations. A homogeneous time‐resolved fluorescence‐based FXR coactivator association assay was conducted to determine the percentage of agonistic and antagonistic activities of the target products at a concentration of 20 μM. Compounds **50** and **51** exhibited the best cellular potencies for agonistic and antagonistic activity, with an EC_50_ of 6.0 ± 0.3 μM and an IC_50_ of 10 ± 1.2 μM, respectively. Compound **51** was therefore selected for further studies on the FXR antagonistic mechanism (Figure 6).

**Figure 7:**
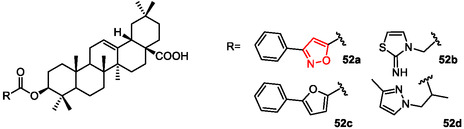
Pentacyclic triterpene‐OA derivatives **52a–52d**.

In 2017, another study proved the chemical modifications at the C‐3 position of the pentacyclic triterpene oleanolic acid (OA) to identify new FXR‐interacting agents.^[^ ^130^ ^]^ Using a 2D fingerprint similarity clustering strategy, the researcher constructed an in silico library of OA derivatives bearing different heterocycle substituents at C‐3. Further docking analysis led to the selection of four top‐scoring OA 3‐O‐ester derivatives for synthesis. Bioassay results showed that all four compounds (**52a‐d**) inhibited chenodeoxycholic acid (CDCA)‐induced FXR transactivation in a concentration‐dependent manner, with compounds **52b** and **52 d** exhibiting greater activity than the parent OA (**Figure** **7**) (IC_50_ = 19.41, 7.03, 13.74, and 9.03 μM, respectively). A molecular simulation study was conducted to explore the structure‐activity relationship (SAR); the antagonistic mechanism and the lower binding affinity of compound **52a**, featured by a 3,5‐disubstituted isoxazole unit, compared to other derivatives and OA, resulted in a lower FXR modulation.

#### 3.1.5 Isoxazole‐Based GPBAR1 Agonists

The studies by Evans et al. (2009) and Budzik et al. (2010) both focused on 3‐aryl‐4‐isoxazolecarboxamides as potent GPBAR1 (TGR5) agonists, aiming to modulate bile acid receptor activity to treat metabolic disorders like type 2 diabetes.

**Figure 8:**
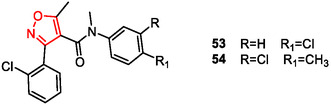
5‐Methyl‐3‐aryl‐4‐isoxazolecarboxamides derivatives **53** and **54**.

The synthesis reported by Evans et al.^[^ ^131^ ^]^ involved the conversion of the commercially available 5‐methyl‐3‐aryl‐4‐isoxazolecarbonyl chlorides into the desired amides in one or two steps. Lead optimization led to the identification of compounds **53** and **54** (**Figure** **8**), which demonstrated strong potency in cellular assays (**53** pEC_50_ 6.8–7.5; **54** pEC_50_ 6.3–7.1). Compound **53** was extensively profiled for selectivity, and it exhibited significant TNFα secretion in monocytes but no notable activity against key CYP450 enzymes. Despite high in vivo clearance and poor systemic exposure, compounds **53** and **54** were considered suitable for targeting GPBAR1 in the gastrointestinal tract via local administration. In an acute conscious dog model, both compounds significantly increased GLP‐1 secretion and reduced portal vein glucose levels, supporting their therapeutic use for metabolic disorders such as type II diabetes and its associated complications.

Budzik et al. described the synthesis and SAR analysis of a series of GPBAR1 agonists,^[^ ^132^ ^]^ identified through high‐throughput screening.

**Figure 9:**
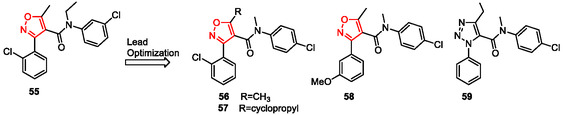
Lead optimization of 5‐Methyl‐3‐aryl‐4‐isoxazole derivative **55** to give derivatives **56**–**59**.

Solution‐phase synthesis enabled rapid analog development, leading to compounds with high potency (up to pEC_5__0_ = 9) and enhanced GLP‐1 secretion in vivo.^[^ ^131^ ^]^ Further optimization of hit compound **55** (pEC_5__0_ = 5.3) led to potent derivatives **56**‐**58** (pEC_50_ = 7.5, 8.4, and 9.0, respectively), while compound **59** (pEC_50_ = 7.9), containing a triazole ring, showed improved in vitro metabolic stability (**Figure** **9**).

### 3.2 Oxadiazole Nucleus

**Figure 10:**
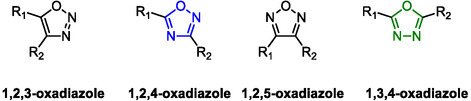
Regioisomers of oxadiazoles.

Oxadiazoles are five‐membered aromatic heterocycles belonging to the azole family. They contain two nitrogen atoms and one oxygen atom and can exist as different regioisomers depending on the arrangement of the heteroatoms within the ring. The main isomers include 1,2,3‐oxadiazole, 1,2,4‐oxadiazole, 1,2,5‐oxadiazole, and 1,3,4‐oxadiazole (**Figure** **10**).

Oxadiazoles and their chemical versatility have strongly increased the curiosity of chemists, particularly in the last decades. Nowadays, these heterocyclic scaffolds have gained importance considering their ability to behave as bioisoster of esters and amides, with several applications in drug discovery and material science. The 1,2,4 and 1,3,4‐oxadiazoles are the most common isomers in medicinal chemistry.

Several studies, based on UV analysis of the bathochromic shift of 3,5‐diphenyl‐oxadiazole derivatives, demonstrated that 1,2,4 isomers exhibit poor aromaticity, whereas the 1,3,4 derivatives show enhanced aromaticity. These two regioisomers also differ in their lipophilic–hydrophilic balance, primarily influenced by their dipole moment. Specifically, 1,2,4‐oxadiazoles show a low level of dipole moment, resulting in more lipophilic compared to the 1,3,4 regioisomers.^[^ ^133^, ^134^ ^]^ These differences are crucial when considering the use of the oxadiazole scaffolds in drug design, as they strongly affect the pharmacokinetic profile, even if the presence of different substituents on the scaffold plays a key role in target modulation.

**Scheme 2:**
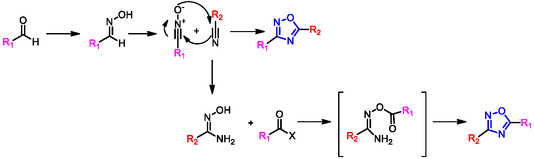
1,3‐Dipolar cycloaddition or Amidoxime route for the preparation of 1,2,4‐oxadiazoles.

The first synthesis of oxadiazoles was reported by Tiemann and Krüger in 1884, initially named furo[ab]diazoles.^[^ ^135^ ^]^ Many 1,2,4‐oxadiazoles possess a general structure with two substituents in positions C‐3 and C‐5. The synthesis of these heterocycles is typically carried out using two most common strategies: the 1,3‐dipolar cycloaddition and the amidoxime route.^[^ ^134^ ^]^ Both strategies require the use of nitrile as a precursor, whose substituent can ultimately be incorporated at different positions on the oxadiazole ring depending on the synthetic route. The selection between these two synthetic approaches is often guided by the commercial availability of the desired precursor, as well as the need to install a specific functional group on the resulting heterocyclic scaffold. The 1,3‐dipolar cycloaddition is a chemical reaction between a 1,3‐dipole and a dipolarophile to give a five‐membered ring, and it is widely used to perform a regio‐ and stereoselective synthesis. The synthesis of 1,2,4‐oxadiazoles through this route can be achieved by cycloaddition between nitrile and nitrile N‐oxide, as shown in **Scheme** **2**. Nitrile N‐oxide is not a commercially available reagent and must be synthesized from the aldehyde, which is first converted to the corresponding oxime by reaction with hydroxylamine, followed by chlorination to lead to the desired nitrile N‐oxide intermediate. Among the synthetic approaches, the amidoxime procedure is generally the most commonly used strategy for the synthesis of 1,2,4‐oxadiazoles (Scheme 2). In this method, the amidoxime intermediate, an oxygen nucleophile, is readily obtained by reacting a nitrile with hydroxylamine.

This intermediate then reacts with an activated carboxylic acid derivative, such as carboxylic acids, esters, anhydrides, acid chlorides, or aldehydes. The reaction proceeds via intramolecular cyclodehydration, forming an O‐acylamidoxime intermediate, which subsequently undergoes heterocyclization to afford the final 1,2,4‐oxadiazole product (Scheme 2).

**Scheme 3:**
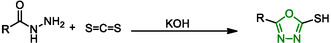
Synthesis of 1,3,4‐oxadiazole building block.

Traditional methods often involve expensive reagents and harsh conditions with organic solvents, posing environmental and economic concerns. To address this, advances in green chemistry have focused on improving sustainability through the use of greener solvents, catalysts, solid supports, and techniques like microwave‐assisted heating.^[^ ^136^ ^]^ One of the most common approaches for the synthesis of the 1,3,4‐oxadiazole scaffold is the reaction of potassium hydroxide (KOH), carbon disulfide (CS_2_), and appropriately substituted acid hydrazide. This approach is typically used to synthesize 2‐mercapto‐5‐aryl‐1,3,4‐oxadiazole (**Scheme** **3**). Its popularity is largely due to the simplicity of the work‐up and the consistently high yields obtained. The lengthy response time, though, is a constraint on the method's ability to produce consistently high yields. To get around this restriction, a number of other synthetic techniques have been documented in the literature.^[^ ^137^ ^]^

#### 3.2.1 Oxadiazole‐Based FXR Agonists

**Figure 11:**
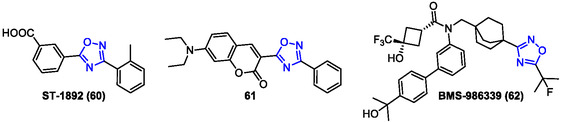
1,2,4‐oxadiazole derivatives **60**–**62**.

Back to FXR modulators, among the oxadiazole derivatives, Flesch et al. reported the discovery of two promising FXR ligands obtained through fragmentation and optimization of the known agonist **GW4064**.^[^ ^43^, ^138^ ^]^ By investigating **GW4064** fragments lacking the aromatic tail, the study identified two key compounds characterized by the presence of an 1,2,4‐oxadiazole ring: a highly potent and soluble partial FXR agonist (compound **60**, **ST‐1892**) and a fluorescent FXR ligand (compound **61**) with potential as a pharmacological tool (**Figure** **11**).

Compound **60** exhibited low nanomolar partial FXR agonistic activity (EC_50_ = 7.2 ± 0.2 nM), making it a promising candidate for further in vitro and in vivo characterization. On the other hand, compound **61** was synthesized by incorporating a coumarin‐derived fluorophore into the central oxadiazole structure. It demonstrated nanomolar partial FXR agonism (EC_50_ = 460 ± 30 nM) and offered practical utility as a fluorescent pharmacological tool for in vitro assays, thanks to its favorable absorption/emission properties, low EC_50_, and straightforward synthesis.

These compounds represented valuable FXR‐targeting agents, with potential both as lead compounds for drug development and as tools for exploring FXR's pharmacological roles.

In 2022, Nara et al. reported the discovery and preclinical pharmacological evaluation of compound **62** (**BMS‐986 339**), a nonbile acid FXR agonist developed through advanced structure‐based drug design (Figure 11). This derivative exhibited a potent in vitro and in vivo FXR activation (FXR EC_50_ = 34 ± 3 nM), though it displayed a context‐dependent profile leading to tissue‐selective effects. As reported, compound **62** showed differential induction of Fgf15 in the liver and ileum through in vivo FXR agonism, demonstrating antifibrotic efficacy despite reduced activation of certain liver‐specific genes. These findings suggested that the distinct FXR liver activation by **BMS‐986 339** does not provide additional efficacy benefits, potentially reducing the risk of adverse effects.^[^ ^139^ ^]^

#### 3.2.2 Oxadiazole‐Based FXR Antagonists

**Figure 12:**
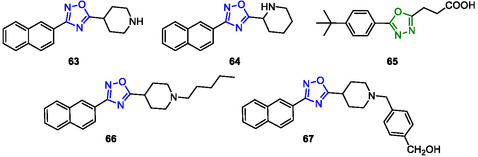
Oxadiazole derivatives (**63–67**) reported in this review.

Recent findings have demonstrated that FXR antagonists may be beneficial for treating cholestasis and related metabolic disorders.^[^ ^56^ ^]^ In 2019, Festa et al. reported the discovery of a new chemotype of FXR antagonists featured by a 3,5‐disubstituted oxadiazole core.^[^ ^140^ ^]^ A total of 35 new derivatives were designed and synthesized, and among them, compounds **63** and **64** (**Figure** **12**), containing a protonable piperidine nucleus at C‐5 and a naphthyl group at C‐3, displayed the best antagonistic activity against FXR with promising cellular potency (IC_50_ 0.58 ± 0.27 and 0.127 ± 0.02 μM, respectively). Compound **63** (3‐(naphthalen‐2‐yl)‐5‐(piperidin‐4‐yl)‐1,2,4‐oxadiazole) showed an impressive ability to counteract the effects of CDCA on the expression of multiple FXR‐targeted genes and, combining its excellent pharmacokinetic properties, resulted in the most promising lead compound, suitable for further development.

Carino et al. demonstrated that compound **63** rescued from negative regulation of the MRP4 gene and provided protection against liver injury in mice induced by treatment with α‐naphthyl‐isothiocyanate (ANIT).^[^ ^141^ ^]^

A novel chemotype of FXR ligands, characterized by a central heterocyclic core, which could be tuned to exhibit either agonistic or antagonistic activity, was described in 2021 by Helmstadter et al.^[^ ^142^ ^]^

In particular, the 1,3,4‐oxadiazole analog **65** (Figure 12) exhibited a sub‐micromolar IC_50_ value of 0.13 ± 0.04 μM for competitive FXR antagonism.

Finamore et al. (2023) introduced a new class of 1,2,4‐oxadiazole derivatives decorating the nitrogen of the piperidine ring with different N‐alkyl and N‐aryl side chains. Their study included the synthesis, pharmacological evaluation, and in vitro pharmacokinetic properties of these derivatives. In vitro pharmacological evaluation showed compounds **66** and **67** as the first examples of nonsteroidal dual FXR/pregnane X receptor (PXR) modulators (**66** FXR IC_50_ = 8.4 ± 2 μM, PXR EC_50_ = 6.4 ± 0.1 μM; **67** FXR IC_50_ = 4.4 ± 0.4 μM, PXR EC_50_ = 7.5 ± 1 μM). In HepG2 cells, these compounds modulated PXR‐ and FXR‐regulated genes, resulting in interesting leads for the treatment of inflammatory disorders (Figure 12).^[^ ^143^ ^]^

#### 3.2.3 Oxadiazole‐Based GPBAR1 Agonists

**Figure 13:**
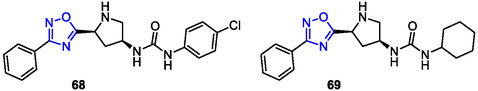
(1,2,4‐oxadiazol‐5‐yl)pyrrolidin‐3‐yl)ureidyl derivatives **68–69**.

In 2019, Di Leva et al. adopted a multidisciplinary strategy combining computational methods, organic synthesis, and pharmacological techniques to design selective agonists for GPBAR1.^[^ ^144^ ^]^ This approach led to the identification of a new class of nonsteroidal agonists, the (1,2,4‐oxadiazol‐5‐yl)pyrrolidin‐3‐yl)ureidyl derivatives. These compounds demonstrated high efficacy and selectivity for GPBAR1. Among them, compounds **68** and **69** (**Figure** **13**) emerged as the most potent (EC_50_ = 3.5 μM (115%) and 4.6 μM (121%), respectively). Notably, compound **69** matched the efficacy of taurolithocholic acid (TLCA), a reference GPBAR1 agonist, inducing the expression of pro‐glucagon mRNA in GLUTag cells. The discovered ligands exhibited favorable pharmacokinetic properties, including good aqueous solubility and high microsomal stability. These findings validate the further exploration of the (1,2,4‐oxadiazol‐5‐yl)pyrrolidin‐3‐yl)ureidyl scaffold for the development of drug candidates targeting GPBAR1‐related disorders.

**Figure 14:**
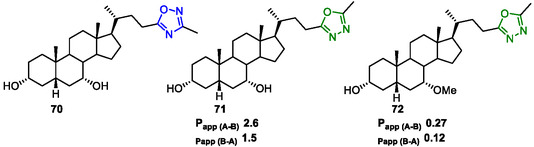
Chenodeoxycholic acid (**CDCA**) derivatives (**70–72**).

In parallel efforts, Nakhi et al. (2019) explored chemical modifications of **CDCA** to optimize GPBAR1 activation.^[^ ^145^ ^]^ By introducing structural variations, particularly at the acid moiety, they significantly improved agonistic potency. The introduction of an oxadiazole core at C‐24 side chain yielded compounds **70** (1,2,4‐oxadiazole derivative) and **71** (1,3,4‐oxadiazole derivative), which exhibited weak and strong GPBAR1 agonistic activities, respectively; the latter being ≈70‐fold more potent than **CDCA** (**Figure** **14**) with an EC_50_ of 8.44 μM for compound **70** and 0.223 μM for compound **71**. Interestingly, methylation of the 7‐hydroxyl group in compound **71** furnished compound **72**, which displayed a highly potent GPBAR1 agonistic activity (EC_50_ = 5.6 ± 0.66 nM). These findings highlight that the substitution of the acid moiety of **CDCA** with a wide variety of chemical groups maintains or even significantly enhances GPBAR1 activation.

The 1,3,4‐oxadiazole derivative **71** and its 7‐methylated analog **72** were also evaluated to identify GPBAR1 agonists with reduced systemic exposure that could serve as suitable treatments for inflammatory bowel disease.

In a mouse model, compound **71** had high oral exposure with a large area under the curve (AUC) and had negligible fecal recovery (<1%). In contrast, oral exposure with derivative **72** was more limited, with an observed AUC of approximately half that of **71** and with greater fecal recovery (7%).^[^ ^146^ ^]^

### 3.3 Oxazole Nucleus

1,3‐Oxazole nucleus represents an important heterocyclic in medicinal chemistry. Several synthetic strategies have been developed to create 1,3‐oxazole derivatives, with a growing emphasis on metal‐free methods that are both efficient and environmentally sustainable.^[^ ^147^ ^]^ These advances continue to expand the role of oxazole derivatives in drug development.

#### 3.3.1 Oxazole‐Based FXR Modulators

**Figure 15:**
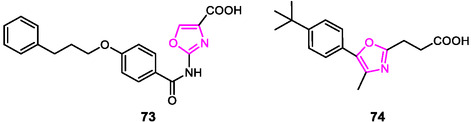
Oxazole‐based FXR modulators.

A directed modulation of multiple targets with a single molecule appears to be a particularly suitable approach for NASH. For this purpose, Schierle et al. (2021) explored the dual activation of the FXRα and the inhibition of leukotriene A4 hydrolase (LTA4Hi), focusing their synthetic modifications on notable FXR/LTA4Hi dual modulators reported in literature.^[^ ^148^ ^]^ Starting with the most balanced sub‐micromolar activity on the two desired targets, they tried to improve its polarity by replacing a pyridine motif with a 1,3‐oxazole group, which lead to compound **73** (**Figure** **15**), but this modification significantly decreases FXR activation, although the result on LTA4Hi remains good (FXR EC_50_ = 11.2 μM, LTA4Hi IC_50_ = 0.16 μM).

Starting from a weak FXR/PPAR agonist, Helmstadter et al. (2021) aimed to expand a library of heterocyclic compounds active as selective FXR modulators, with nanomolar to low‐micromolar potencies and binding affinities, in order to achieve new, potentially experimental drugs.^[^ ^142^ ^]^

The introduction of a 4‐methyloxazole nucleus in their synthesized library led to a loss of FXR activation and resulted in the identification of a weak FXR antagonist (compound **74**, IC_50_ = 13.9 μM, Figure 15).

## 4 Summary and Outlook

Five‐membered heteroaromatic rings have attracted significant attention in medicinal chemistry due to their favorable properties, including enhanced metabolic stability, solubility, and bioavailability, key attributes in the development of effective drugs.

This review provides an overview of five‐membered aromatic heterocycles containing nitrogen and oxygen atoms that are capable of modulating BARs. Particular emphasis is placed on compounds active toward FXR and GPBAR1, two receptors that have emerged as promising targets in drug discovery.

BARs play a pivotal role in regulating various physiological and inflammatory pathways. They form a heterogeneous family of receptors activated by bile acids and are implicated in several hepatic and metabolic disorders, including liver fibrosis, diabetes, and NASH.

A structure–activity relationship (SAR) analysis drawn from the literature highlights the crucial influence of the heterocyclic moiety on pharmacological activity. In particular, the introduction of an isoxazole ring into nonsteroidal small molecules has led to the development of FXR agonists. Since the discovery of **GW4064** in 2006, most synthesized FXR agonists have featured a 3,4,5‐trisubstituted isoxazole core. In contrast, only a few examples of 3,5‐disubstituted isoxazoles, either as agonists or antagonists, have been reported, and these generally exhibit weak modulatory activity. Moreover, introducing bulkier substituents at C‐5, larger than cyclopropyl or isopropyl, or replacing the isoxazole with an oxadiazole nucleus has been associated with a complete shift from FXR agonism to antagonism.

The incorporation of an oxadiazole ring, characterized by distinct geometry and electronic properties due to the presence of an additional N heteroatom, has led to the development of several modulators exhibiting a broad spectrum of activity toward FXR and/or GPBAR1. The rigidity of the oxadiazole ring limits the conformational flexibility required for FXR activation. However, in GPBAR1, the more flexible and permissive binding pocket facilitates the agonistic modulation. The additional nitrogen atom can engage favorable hydrogen bonds or polar interactions with key residues in the GPBAR1 binding site, promoting receptor activation.

Conversely, the incorporation of an oxazole nucleus has generally resulted in poor FXR modulation, indicating its limited suitability for strong receptor interaction in this context.

Based on these considerations, the aim of this review is to provide an in‐depth look at the literature, from early studies to the most recent findings. This serves as a valuable resource for researchers, offering guidance in the design and development of novel and successful lead compounds targeting BARs.

## Conflict of Interest

The authors declare no conflict of interest.
